# Waning effectiveness of the third dose of the BNT162b2 mRNA COVID-19 vaccine

**DOI:** 10.1038/s41467-022-30884-6

**Published:** 2022-06-09

**Authors:** Tal Patalon, Yaki Saciuk, Asaf Peretz, Galit Perez, Yoav Lurie, Yasmin Maor, Sivan Gazit

**Affiliations:** 1 Kahn Sagol Maccabi (KSM) Research & Innovation Center, Maccabi Healthcare Services, Tel Aviv, 68125 Israel; 2 Maccabitech Institute for Research and Innovation, Maccabi Healthcare Services, Tel Aviv, Israel; 3 Internal Medicine COVID-19 Ward, Samson Assuta Ashdod University Hospital, Ashdod, Israel; 4 Liver unit, Shaare Zedek City Center Campus, Jerusalem, Israel; 5 Faculty of Medicine, Tel Aviv University, Tel Aviv, Israel; 6 Infectious Disease Unit, Edith Wolfson Medical Centre, Holon, Israel

**Keywords:** Epidemiology, Public health, Vaccines, SARS-CoV-2

## Abstract

The duration of protection of the third (booster) dose of the BioNTech/Pfizer BNT162b2 mRNA Coronavirus Disease 2019 vaccine has been the subject of recent investigations, as global discussions around the necessity and effectiveness of a fourth dose are already underway. By conducting a retrospective study implementing a test-negative case-control design, analyzing 546,924 PCR tests performed throughout January 2022 by 389,265 persons who received at least two doses, we find that the effectiveness in each month-since-vaccination decreases significantly. Compared to those vaccinated five months prior to the outcome period, on August 2021, relative protection against infection waned from 53.4% a month after vaccination to 16.5% three months after vaccination. These results suggest that there is a significant waning of vaccine effectiveness against the Omicron variant of the third dose of the BNT162b2 vaccine within a few months after administration. Additional information could assist to comprehensively estimate the effectiveness of the three-dose-strategy.

## Abstract (web summary)

In this retrospective study, authors show that relative protection against SARS-CoV-2 infection wanes from 53.4% one month after vaccination to 16.5% three months after vaccination, suggesting that there is a significant waning of mRNA vaccine effectiveness against infection with the Omicron variant.

## Introduction

Both the short-term effectiveness of two doses of the BioNTech/Pfizer BNT162b2 mRNA coronavirus disease 2019 (COVID-19) vaccine^1–4^ and the waning of the vaccine-induced immunity have been demonstrated in previous research^5–9^, though waning has been mild against severe disease^10^. These studies, alongside the surge of breakthrough infections with the Delta Variant (B.1.617.2) of the SARS-CoV-2 led policymakers around the globe to administer the third (booster) dose of the vaccine.

In Israel, an early adopter of the two-dose BNT162b2 regimen, a similarly rapid campaign was carried out for the third dose. The booster was first approved on July 30, 2021 for all individuals aged 60 or older who received the second dose at least five months prior, and by August 29, 2021, the campaign was extended to include all individuals aged 12 or older. This swift rollout of the booster facilitated early real-world investigations of its short term effectiveness, largely demonstrating a restored short-term effectiveness^7,9,11,12^.

However, initial evidence have begun to surface as to the duration of protection of the third dose and its effectiveness against different variants, most recently initial evidence waning effect against Delta and Omicron variants^13^. In Israel, the recent surge of infections with the highly transmissible Omicron (B.1.1.529)^14,15^ variant has led to the January 2, 2022, official recommendation to administer a fourth dose of the vaccine to medical personnel and people ages 60 and older^16^, while expanding the eligibility criteria to immunocompromised individuals aged 18 or older^17,18^.

To this end, we conducted a retrospective analysis examining the association between booster breakthrough infections with the Omicron variant and time-since-vaccination, leveraging data from Maccabi Healthcare Services (MHS), an Israeli health fund that covers 2.5 million lives.

## Results

During the outcome period of January 1 to January 21, 2022, 546,924 PCR tests by 389,265 eligible MHS members were performed. From the main analysis, 53,486 tests (performed by 38,145 individuals) were excluded, as they were conducted after the administration of the fourth dose (see additional analyses). Population characteristics can be found in Table 1. Overall, those vaccinated early were more chronically ill, likely correlating with earlier compliance to vaccination by the older population, emphasizing the need for adjustment.

**Table 1:** Population characteristics by exposure groups in tested population.

	Exposure Groups/Booster period						
	Second dose only	Aug 2021	Sep 2021	Oct 2021	Nov 2021	Dec 2021	Overall
	*(N* = *54221)*	*(N* = *129868)*	*(N* = *142238)*	*(N* = *47515)*	*(N* = *8233)*	*(N* = *7190)*	*(N* = *389265)*
**Sex**							
Female	32,363 (59.7%)	71,664 (55.2%)	82,343 (57.9%)	29,308 (61.7%)	4959 (60.2%)	4260 (59.2%)	22,4897 (57.8%)
Male	21,858 (40.3%)	58,204 (44.8%)	59,895 (42.1%)	18,207 (38.3%)	3274 (39.8%)	2930 (40.8%)	16,4368 (42.2%)
**Age Groups**							
[16, 30)	22,633 (41.7%)	1568 (1.2%)	51,574 (36.3%)	18,965 (39.9%)	3428 (41.6%)	3569 (49.6%)	10,1737 (26.1%)
[30, 40)	12,327 (22.7%)	5889 (4.5%)	37,780 (26.6%)	11,873 (25.0%)	2103 (25.5%)	1593 (22.2%)	71,565 (18.4%)
[40, 50)	9458 (17.4%)	25,694 (19.8%)	32,911 (23.1%)	8905 (18.7%)	1365 (16.6%)	1023 (14.2%)	79,356 (20.4%)
[50, 60)	5463 (10.1%)	42,065 (32.4%)	13,667 (9.6%)	4835 (10.2%)	662 (8.0%)	547 (7.6%)	67,239 (17.3%)
60+	4340 (8.0%)	54,652 (42.1%)	6306 (4.4%)	2937 (6.2%)	675 (8.2%)	458 (6.4%)	69,368 (17.8%)
**Socioeconomic status**							
High	12,622 (23.3%)	54,111 (41.7%)	58,723 (41.3%)	13,315 (28.0%)	2429 (29.5%)	2312 (32.2%)	14,3512 (36.9%)
Middle	29,689 (54.8%)	62,546 (48.2%)	69,872 (49.1%)	26,750 (56.3%)	4348 (52.8%)	3712 (51.6%)	19,6917 (50.6%)
Low	11,831 (21.8%)	12,978 (10.0%)	13,468 (9.5%)	7378 (15.5%)	1444 (17.5%)	1157 (16.1%)	48,256 (12.4%)
Missing	79 (0.1%)	233 (0.2%)	175 (0.1%)	72 (0.2%)	12 (0.1%)	9 (0.1%)	580 (0.1%)
**Social Sector**							
General Jewish	46,446 (85.7%)	123,210 (94.9%)	134,380 (94.5%)	43,210 (90.9%)	7352 (89.3%)	6410 (89.2%)	361,008 (92.7%)
Arab	4243 (7.8%)	2610 (2.0%)	3904 (2.7%)	2717 (5.7%)	597 (7.3%)	494 (6.9%)	14,565 (3.7%)
Ultra-Orthodox Jew	3532 (6.5%)	4048 (3.1%)	3954 (2.8%)	1588 (3.3%)	284 (3.4%)	286 (4.0%)	13,692 (3.5%)
**Cardiovascular diseases**							
No	51,886 (95.7%)	111,880 (86.1%)	137,876 (96.9%)	45,952 (96.7%)	7899 (95.9%)	6936 (96.5%)	362,429 (93.1%)
Yes	2335 (4.3%)	17988 (13.9%)	4362 (3.1%)	1563 (3.3%)	334 (4.1%)	254 (3.5%)	26,836 (6.9%)
**Diabetes Mellitus**							
No	52,315 (96.5%)	110,743 (85.3%)	138,150 (97.1%)	46,073 (97.0%)	7940 (96.4%)	6992 (97.2%)	362,213 (93.1%)
Yes	1906 (3.5%)	19,125 (14.7%)	4088 (2.9%)	1442 (3.0%)	293 (3.6%)	198 (2.8%)	27,052 (6.9%)
**Hypertension**							
No	49,678 (91.6%)	87,595 (67.4%)	132,847 (93.4%)	43,901 (92.4%)	7538 (91.6%)	6725 (93.5%)	328,284 (84.3%)
Yes	4543 (8.4%)	42,273 (32.6%)	9391 (6.6%)	3614 (7.6%)	695 (8.4%)	465 (6.5%)	60,981 (15.7%)
**CKD**							
No	52,108 (96.1%)	106,643 (82.1%)	138,241 (97.2%)	45,987 (96.8%)	7888 (95.8%)	6990 (97.2%)	357,857 (91.9%)
Yes	2113 (3.9%)	23,225 (17.9%)	3997 (2.8%)	1528 (3.2%)	345 (4.2%)	200 (2.8%)	31,408 (8.1%)
**COPD**							
No	53,860 (99.3%)	125,985 (97.0%)	141,682 (99.6%)	47,266 (99.5%)	8176 (99.3%)	7161 (99.6%)	384,130 (98.7%)
Yes	361 (0.7%)	3883 (3.0%)	556 (0.4%)	249 (0.5%)	57 (0.7%)	29 (0.4%)	5135 (1.3%)
**Obesity (BMI** ≥ **30)**							
No	47,998 (88.5%)	102,774 (79.1%)	125,861 (88.5%)	41,762 (87.9%)	7298 (88.6%)	6465 (89.9%)	332,158 (85.3%)
Yes	6223 (11.5%)	27,094 (20.9%)	16,377 (11.5%)	5753 (12.1%)	935 (11.4%)	725 (10.1%)	57,107 (14.7%)
**Immunosuppression**							
No	52,556 (96.9%)	118,570 (91.3%)	138,071 (97.1%)	46,233 (97.3%)	7943 (96.5%)	7000 (97.4%)	370,373 (95.1%)
Yes	1665 (3.1%)	11,298 (8.7%)	4167 (2.9%)	1282 (2.7%)	290 (3.5%)	190 (2.6%)	18,892 (4.9%)

*CKD* Chronic Kidney Disease, *COPD* Chronic Obstructive Pulmonary Disease.

**Fig. 1:**
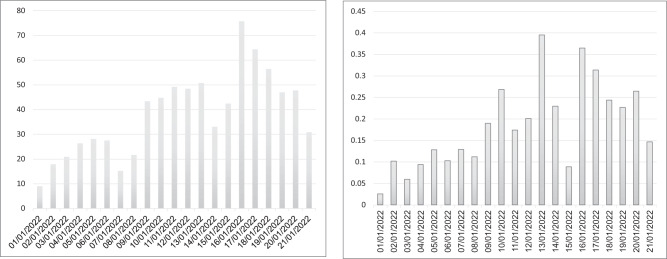
Daily SARS-Cov-2 infection and SARS-Cov-2-related hospitalization rates per 10,000 persons. The graph exemplifies Omicron’s spread, with a rapid increase in infections and hospitalizations, peaking in mid-January.

The follow-up period exemplified Omicron’s rapid spread, with an increase in the daily numbers of infections and severe disease (Fig. 1). During the examined Omicron-dominant period, a total of 101,737 breakthrough infections and 482 breakthrough infections resulting in COVID-19 hospitalizations or deaths were detected, of which 30,870 infections, 208 hospitalizations and 9 deaths were among those who received the booster dose early, in August 2021, whereas 1,082 infections, 4 hospitalizations and zero mortality were among the ‘newly vaccinated’ booster recipients, receiving their booster dose in December 2021. In that same Omicron period, 16,938 infections and 122 hospitalizations were recorded among those who received only two doses.

### Main analysis–test-negative case-control design

Table 2 details the results of the conditional regression in terms of the derived VE, whereas table S1 details the adjusted Odds Ratio (aOR) in each month-from-vaccination. The aOR represents the ratio between the odds of testing positive or having severe COVID-19 during the January 1-21, 2022 period among those vaccinated in August 2021 (the first month of eligibility to the booster shot) and the respective odds in each subsequent month of vaccination.

**Table 2:** Main analysis; adjusted Third Dose Effectiveness (VE) against SARS-Cov2 infection.

Exposure Groups/Booster Period	Cases	Controls	Adjusted Third Dose VE (%) for SARS-Cov2 Infection (CI 95%)
Aug. 2021 (−5)‡	21,777	21,234	Reference
Sep. 2021 (−4)‡	25,419	24,432	3.6 (0.6, 6.5)
Oct. 2021 (−3)‡	7050	7773	16.5(13, 19.9)
Nov. 2021 (−2)‡	792	1156	35.7(29.8, 41.2)
Dec. 2021 (−1)‡	421	864	53.4(47.7, 58.6)
	55459	55459	

‡ Denotes the months prior to the outcome period. Therefore, those who received the booster in October 2021, received the third dose 3 months prior to the outcome period.

VE was defined as 100%*[1-(Odds Ratio)] for each of the month-since-vaccination categories.

ORs were estimated with conditional logistic regression on a 1:1 matched dataset, and adjusted for comorbidities.

**Fig. 2:**
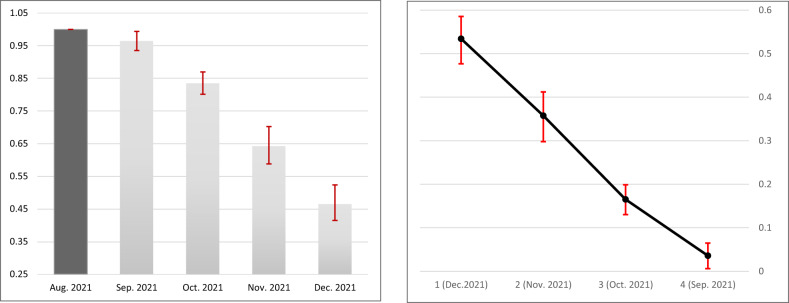
Adjusted odds ratio (left) and vaccine effectiveness (right) against SARS-CoV-2 breakthrough infection. Left: Data are presented as adjusted OR, as a function of time in months (calendrical month) since the administration of the third dose, with error bars indicating the corresponding 95% Wald’s C.I. Right: Data are presented as adjusted VE, as a function of time in months (calendrical month) since the administration of the third dose, with error bars indicating the corresponding 95% Wald’s C.I.

In the main analysis, we matched 110,918 cases and controls (in a 1:1 ratio) for the breakthrough infection analysis, and 562 cases and controls for the severe COVID-19 analysis (hospitalization or death). The vaccine effectiveness against infection of ‘newly’ vaccinated individuals (i.e. those receiving the booster in December, a month prior to the outcome period in January 2022) was 53.4% (95% Confidence Interval [CI], 47.7–8.6%) compared to those vaccinated early, in August. VE declined rapidly with each month since vaccination, with 35.7% (95% CI, 29.8–41.2%) VE for those who were vaccinated two months prior to the outcome period (i.e. in November 2021) compared to the earliest vaccinees (of August 2021). Following the same pattern, VE was 16.5% (%95 CI, 13.0–19.9%) for the those who were vaccinated *three* months prior to the outcome period (i.e., October 2021 vaccinees), compared to the earliest vaccinees. Those who received the booster shot four months prior to the outcome period, in September 2021, had comparable odds of infection to those receiving the booster a month earlier, in August 2021 (Table 2 and Fig. 2). None of the comorbidities were significantly related to the odds of testing positive **(**Table S1). Finally, the extent to which each comorbidity modified the VE can be seen in Table S3. Significant effect modification was found only for obesity, with lower odds ratio in obese individuals for whom more than a month-since-inoculation had passed prior to the outcome period (likelihood ratio test *p* < 0.01). We did not find effect modification for any of the other comorbidities.

As for severe disease, we did not reach significance implying a decreasing VE against hospitalizations or death as a function of time-since-inoculation (Table 2 and S1). However, the raw numbers are too small to support a reliable conclusion (Table S2).

### Additional Analyses

The results of the first sensitivity analysis (allowing for individuals to contribute multiple negative tests using a GEE logistic regression model yielded estimates of VE that were overall similar to the results presented in the main analysis (table S4**)**. As we observed higher ORs when the second dose was given early, namely in February and March 2021 (which was not evident when the second dose was administered in later months, closer to the administration of the third dose) we subsequently attempted to measure the interaction between time-since-second-dose and time-since-booster. However, the numbers were too small to run such an analysis. Therefore, we created 3/4-months intervals for the timing of the second dose (January through March 2021, versus April to July 2021). Nonetheless, none of these interactions were significant.

The second sensitivity analysis, which incorporated tests performed on days 0-6 after the fourth vaccine dose, yielded overall similar results **(**table S5). Nonetheless, when analyzing tests performed on days 0-6 after the fourth dose as a separate time-since-vaccination stratum, we found that VE during this interval was 28.9% (95% CI, 24.4–33.1%) (table S6).

The third sensitivity analysis included changing the reference group; instead of a comparison within the booster recipients (that is, comparing the earlier vaccinees to late vaccinees), we compared each month of booster vaccinees to persons unvaccinated with the booster, namely those who received only two doses. Thus, we estimated the marginal effectiveness, or the added benefit of the booster compared to the second dose. In this analysis, we matched 128,854 persons for the breakthrough infection analysis and 722 persons for the severe COVID-19 analysis (hospitalization or death). The marginal effectiveness against infection of a booster dose given a month before the outcome period was at its peak at 59.4% (95% CI, 54.9–63.5%). Effectiveness declined gradually with time from inoculation, reaching 16% (95% CI, 12.3–19.5%) in those vaccinated 5 months prior to the outcome period compared to those not receiving the booster dose (Table 3 and S7). As for the marginal effectiveness against severe disease, it seems that waning exists though to a much lesser degree, as effectiveness declines from 72.2% (95% CI, 37.8–87.6%) 3 months after inoculation to 54.5% (95% CI, 13.4–76.1) five months after vaccination. However, numbers are small as also reflected by the confidence intervals.

**Table 3:** Additional analysis; adjusted Third Dose Effectiveness (VE) against SARS-Cov2 infection, compared to second-dose only vaccinees.

Exposure Groups/Booster Period	Cases	Controls	Adjusted Third Dose VE (%) for SARS-Cov2 Infection (CI 95%)
Second Dose Only	7866	6264	Reference
August 2021 (−5)‡	21,900	21,588	16 (12.3, 19.5)
September 2021 (−4)‡	25,920	25,623	18.3 (15.2, 21.2)
October 2021 (−3)‡	7378	8583	29.1 (26.1, 32)
November 2021 (−2)‡	872	1305	43.2 (38.2, 47.8)
December 2021 (−1)‡	491	1064	59.4 (54.9, 63.5)
	64,427	64,427	

‡ Denotes the months prior to the outcome period. Therefore, those who received the booster in October 2021, received the third dose 3 months prior to the outcome period.

Additionally, including in the analysis individuals with hybrid immunity, i.e., those infected prior to August 2021, yielded similar results to our main analysis (Table S8). Nonetheless, as can be seen in the regression analysis, prior infection provides protection against reinfection (Table S9).

Finally, evaluating the representativeness of the control group (Table S10), suggests that the case-control test-negative design is an appropriate design for this research question.

## Discussion

This research investigated the long-term duration of protection of the third (booster) dose of the BioNTech/Pfizer mRNA BNT162b2 vaccine against breakthrough infections largely due to the Omicron variant of SARS-CoV-2; a pivotal question in light of global discussions about the need for a fourth vaccine dose.

Our main analysis took a test-negative approach, comparing VE between recipients of the booster at different time points from inoculation. The analysis is based on the premise that had there been no waning of the booster protection over time, we would have seen no difference in infection odds at different times from vaccination (within the same calendrical outcome period). Nonetheless, we found that the effectiveness in each month since vaccination decreased significantly, whereas VE against infection compared to the first month of eligibility of the booster (August 2021) declined from 53.4% a month after vaccination to 16.5% three months after the booster dose. As for marginal protection of the third dose compared to the second dose (that is, protection gained on top of the one gained from two doses), we found a residual vaccine effectiveness of 16% after 5 months. Though the data suggests a milder waning of protection against severe disease, the number of hospitalizations and mortality were too small to reach a reliable conclusion.

Studies have demonstrated that the third dose increases immunogenicity against SARS-CoV-2 as reflected by a rapid and broad immune response to the third BNT162b2 dose^7,11^. Our results suggested a time-dependent response with a primary increase in VE followed by waning of effectiveness. Overall, this waning of protection matches our knowledge of the course of the second dose of the BNT162b2, where protection increases immediately after a dose, but decreases rapidly within a few months. However, it seems that effectiveness could be declining faster than that of the second dose^6,19^. The faster decline might partly be explain by an escape of the Omicron variant from neutralizing antibody responses^20^, though further variant-specific evidence need to be established. Our results further point to the fact that waning of VE is not primarily confounded by comorbidities, but rather that the most important factor in long-term VE is time from the last inoculation.

This study has some important limitations. The main limitation of every observational SARS-CoV-2 study is a potential bias stemming from healthcare-related behavior as it pertains to PCR testing. This issue has been discussed in previous studies as well^5,7,11^, and was more of a concern during the past months, in light of changing testing policies in Israel during the Omicron surge, where an overwhelming numbers of infected individuals did not allow the same test-for-all policy, implemented in previous waves. The test-negative design, which does not assume that a lack of a positive test equals a negative test and somewhat adjusts for testing volume, attempts to better mitigate this bias and is the reason we chose this method for this analysis, especially Additionally, the August 2021 vaccinees, who served as the comparison group in the main analysis were generally older and correspondingly sicker. This limitation has also been discussed in previous COVID research, as vaccination rollout generally prioritized chronically ill individuals. Nonetheless, our matching and adjustment approach renders residual confounding by indication less likely, though effect modification can be seen in obesity, potentially explained by self-preservation behavior of a publicized high-risk group through nationwide campaigns. Furthermore, as the Omicron variant was the dominant variant in Israel during the analysis, the waning effect against other strains could not be evaluated. This could explain that in our secondary analysis of the marginal effectiveness, the additional benefit gained one month after inoculation was lower than the VE demonstrated in the same time frame in a previous research^11^, investigating the Delta variant. Lastly, our database does not include reason for being tested, which potentially could differ between time-since-vaccination groups and potentially influence a priori outcome probabilities, a possible confounder which could not be adjusted for.

In conclusion, this study demonstrates significant waning of VE of the third dose of the BNT162b2 vaccine against infection within a few months after administration, though significance was not reached as to a milder waning effect against severe disease, probably due to limited numbers. This waning effect against infection should prompt policy discussion as to vaccine development and future dose allocation, particularly to low-risk populations. Additional information could assist to comprehensively estimate the effectiveness of the three-dose strategy.

## Methods

### Study population and data sources

The study population included all MHS members aged 16 or older who received at least two doses of the BNT162b2 vaccine by August 1, 2021. Individuals were excluded from the study if they had a positive SARS-CoV-2 polymerase chain reaction (PCR) assay test result prior to the start of the outcome period, disengaged from MHS for any reason prior to the start of the study period or joined MHS after to March 2020, hence might have an incomplete COVID-19-related history. Anonymized Electronic Medical Records (EMRs) were retrieved from MHS’ centralized computerized database, a state-mandated, non-for-profit, health fund in Israel which covers 26.7% of the population. This centralized database has been maintained for over three decades, allowing for a comprehensive longitudinal medical follow-up, including demographic data, clinical measurements, outpatient and hospital diagnoses and procedures, medications dispensed, imaging performed and comprehensive laboratory data from a single central laboratory.

### Data extraction

Individual-level demographic data of the study population included sex, age and a coded residential socioeconomic geographical statistical area (GSA), assigned by Israel’s Central Bureau of Statistics, which is the smallest geostatistical unit of the Israeli census (corresponds to neighborhoods) and embodies the socioeconomic status. COVID-19 related data consisted of dates of vaccination and results of any polymerase chain reaction (PCR) tests for SARS-CoV-2, given that all such tests are recorded centrally. PCR tests were readily available during the outcome period, for reasons including clinical symptoms, suspected exposure to infected individuals, public events requirements and more. COVID-19-related hospitalizations and mortality records were retrieved as well. Captured data also consisted of information on chronic diseases from MHS’ automated registries, including cardiovascular diseases^21^, hypertension, diabetes^22^, chronic kidney disease (CKD)^23^, chronic obstructive pulmonary disease (COPD), obesity (defined as a body mass index of 30 or higher) and immunocompromised conditions.

### Measured outcomes

We evaluated two SARS-CoV-2-related outcomes. The first was breakthrough infections, defined as a positive documented RT-PCR test 7 days or more after a vaccine dose (i.e., a booster breakthrough infection was defined as a positive test taken 7 or more days after inoculation and not after the fourth dose, if such was administered). The 7-day cutoff was chosen based on previous research the BNT162b2 vaccine effectivenesss^1,11^. The second outcome referred to a severe disease, defined as a COVID-19-related hospitalization or death occurring within the same time frame. Outcomes were evaluated during the follow-up period of January 1 to January 21, 2022, an Omicron-dominant period in Israel^14^.

The analysis was stratified to different groups, each stratum represented a different time-since-vaccination. The rationale is that if no waning of the booster exists, the rates of breakthrough infection will not depend on the time-from-vaccination. However, if waning of the booster does occur, protection conferred by the third dose will gradually decrease with time-since-vaccination.

### Design and statistical analysis

#### Main analysis–test-negative case-control design

Our main analysis consisted of a test-negative case-control design. The test-negative design, which has long been used in vaccine effectiveness research, is becoming increasingly common in SARS-CoV-2 vaccine studies, due to its strength in controlling against bias stemming from healthcare-seeking behavior^11,24,25^. In this analysis, we defined cases as PCR-positive persons or persons with a severe manifestation of COVID-19 during the follow-up period (cases were defined for each of the two outcomes separately). Eligible controls were PCR negative individuals who had neither tested positive prior to the date of the positive PCR or the COVID-related hospitalization of their matched case and whose test was not done after administration of the fourth vaccine, if such was administered. A 1:1 matching was performed based on sex, age group (16 to 29 years, 30 to 39 years, 40 to 49 years, 50 to 59 years, and ≥60 years), GSA, calendrical week of testing, and the month of receipt of the second dose. The first positive PCR test and the first negative PCR test were the only tests included for each case and control, respectively. All negative PCRs for cases were excluded from the study, therefore an MHS member was either a case or a control, not both^6^.

The analysis sought to estimate the reduction in the odds of a positive outcome at different time points following the receipt of the booster dose. Therefore, we stratified the analysis for each month-since-vaccination, where in each stratum matched pairs included only cases that met that specific month since vaccination or the reference group of August 2021vaccinees (hence, the number of matched pairs differed between time points)^6^. The vaccination status (and time from inoculation) was determined at the time of the PCR test (for the first outcome assessing breakthrough infections) or the COVID-19 hospitalization (for the second outcome assessing severe disease).

A conditional logistic regression model was fit to the data, accounting for the matching. The vaccine effectiveness (VE) of the booster in each month, compared to the first month of eligibility for the booster^19^ (August 2021) was calculated as 100%*[1-(Odds Ratio)] for each of the month-since-vaccination categories. To address potential confounders, we adjusted for underlying comorbidities, including obesity, cardiovascular diseases, diabetes, hypertension, chronic kidney disease, COPD and immunosuppression conditions. Furthermore, we conducted a separate analysis, fitting a series of regression analyses to assess potential effect modification by each of these comorbidities.

#### Additional analyses

We carried out several additional analyses. First, as a sensitivity analysis for the occurrence of a booster breakthrough infection, we repeated the test-negative analysis, this time unmatched allowing individuals to contribute multiple negative tests, but excluding them once they tested positive (further details can be found in Patalon et al.)^11^. In order to account for repeated sample collection from the same individual, a Generalized Estimating Equation (GEE) logistic regression model was fit to the data^26^. Adjustment included demographics and the same comorbidities as the main analysis, in addition to a series of dummy variables representing the calendar week in which the PCR test was performed to address exposure. We assumed an exchangeable correlation structure. In our second sensitivity analysis, we reran the main analysis, while, including tests that were performed 0 to 6 days after the administration of the fourth dose of the vaccine. The rationale was that if we consider ‘fully vaccinated’ individuals as those 7 days or more after receiving a fourth dose (similarly to the booster), tests performed within that period should not be excluded. This also allowed us to mitigate a potential selection bias of excluding tests of early vaccinees. Following this analysis, we attempted to specifically examine days 0-6, by carrying out an additional analysis addressing this interval as a separate time-since-vaccination stratum.

In the third sensitivity analysis, we performed the main analysis again while changing the reference group; instead of the earliest booster vaccinees (i.e., those vaccinated in August 2021), we compared each stratum of time-since-booster to those unvaccinated with the booster, namely persons who received *only* two doses of the vaccine. The advantage of such an analysis is the ability to assess whether the *marginal* effectiveness of the booster over the second dose decreases with time. However, as by the outcome period of January 2022 most of Israel’s population had already received the booster, the reference group of those unvaccinated is admittedly prone to bias, as has been discussed in the previous papers^11^. Therefore, in light of this concern, we chose to present this analysis as a supplementary one.

In the fourth additional analysis, we repeated the main analysis, this time including individuals with hybrid immunity^27^ as well, meaning those who were infected with SARS-CoV-2 prior to August 2021 *and* received three doses of the BNT162b2 vaccine. Notably, this interval meets the minimum 90-day-interval required to comply with a re-infection definition, as defined by the Centers for Disease Control and Prevention^28^.

Finally, in order to validate the underlying assumption of the case-control test-negative design, according to which the control group should mirror particularly the exposure of the source population, we presented the distribution of time since vaccination in the control group versus the source population, i.e. the entirety of the MHS eligible population.

All analyses were performed using R Studio version 3.6 with the MatchIt, gee and survival packages. The analysis conforms to Strengthening the Reporting of Observational Studies in Epidemiology (STROBE) Statement.

### Reporting summary

Further information on research design is available in the Nature Research Reporting Summary linked to this article.

## Supplementary information

Supplementary Information

Reporting Summary

## Data Availability

According to the Israel Ministry of Health regulations, individual-level data cannot be shared openly. Specific requests for remote access to de-identified community-level data should be referred to KSM, Maccabi Healthcare Services Research and Innovation Center.

## Code availability

Specific requests for remote access to the code used for data analysis should be referred to KSM, Maccabi Healthcare Services Research and Innovation Center.
