# Supplementary material for: Waning effectiveness of the third dose of the BNT162b2 mRNA COVID-19 vaccine
Source: Nat Commun. 2022 Jun 9;13:3203. doi: 10.1038/s41467-022-30884-6 (PMC9184525; doi:10.1038/s41467-022-30884-6)
Supplement: Supplementary file 1 — Supplementary Information [file 41467_2022_30884_MOESM1_ESM.pdf]

Waning Effectiveness of the Third Dose of the BNT162b2 mRNA COVID-19 Vaccine

Supplementary Tables

Tal Patalon, MD[1,2]*; Yaki Saciuk, MPH MA[1]; Asaf Peretz, MD[2,3]; Galit Perez, MN MA[2]; Yoav Lurie[4]; Yasmin Maor, MD[5,6]; Sivan Gazit, MD MA[1,2]

*Corresponding author.

[1]Kahn Sagol Maccabi (KSM) Research & Innovation Center, Maccabi Healthcare Services, Tel Aviv, 68125, Israel.

[2] Maccabitech Institute for Research and Innovation, Maccabi Healthcare Services, Israel.

[3]Internal Medicine COVID-19 Ward, Samson Assuta Ashdod University Hospital, Ashdod Israel.

[4]Liver unit, Shaare Zedek City Center Campus, Jerusalem, Israel.

[5]Faculty of Medicine, Tel Aviv University, Tel Aviv, Israel.

[6]Infectious Disease Unit, Edith Wolfson Medical Centre, Holon, Israel.

Table S1. Conditional logistic regression results for SARS-Cov2 infection and COVID-19-related hospitalization.

	**Infection**		**SARS-Cov2 Related Hospitalization**	
	OR	*CI(95%)*	*OR*	*CI(95%)*
Exposure Groups/Booster period(2)				
Sep. 2021 (-4)‡	0.964	(0.935, 0.994)	0.876	(0.477, 1.607)
Oct. 2021 (-3)‡	0.835	(0.801, 0.87)	0.812	(0.345, 1.911)
Nov. 2021 (-2)‡	0.643	(0.588, 0.702)	0.672	(0.075, 6.052)
Dec. 2021 (-1)‡	0.466	(0.414, 0.523)	2.681	(0.321, 22.383)
Cardiovascular Diseases	0.964	(0.919, 1.012)	1.611	(0.928, 2.798)
Diabetes Mellitus	0.945	(0.903, 0.989)	0.795	(0.467, 1.354)
Hypertension	0.994	(0.96, 1.029)	2.448	(1.496, 4.005)
COPD	0.890	(0.808, 0.98)	2.354	(0.97, 5.71)
Immunosuppression	0.949	(0.901, 0.998)	3.441	(1.878, 6.305)
Obesity (BMI ≥30)	0.946	(0.918, 0.976)	0.915	(0.566, 1.481)

In the analysis of VE against infection, the number of cases was 55,459 and controls 55,459.
In the analysis of VE against SARS-Cov2 related hospitalization, the number of Cases was 281 and Controls 281
(2) Reference period: administration of booster during August 2021 (5 months prior to the outcome period)
COPD: Chronic Obstructive Pulmonary Disease ‡ Denotes the months prior to the outcome period. Therefore, those who received the booster in October 2021, received the third dose 3 months prior to the outcome period.

Table S2. Cases and Controls of the severe COVID-19 matched analysis.

Exposure Groups/Booster Period	Hospitalizations (Cases)	Controls
Second Dose Only	70	43
Aug. 2021	195	197
Sep. 2021	74	81
Oct. 2021	17	33
Nov. 2021	3	5
Dec. 2021	2	2
	361	361

Table S3. Effect modification of VE by the different comorbidities.

Exposure Groups/Booster period(1)	Cardiovascular Diseases	Diabetes Mellitus	Hypertension	COPD	Immunosuppression	Obesity (BMI ≥30)
Sep. 2021 (-4)‡	0.97 (0.94, 1)	0.97 (0.94, 1)	0.97 (0.94, 1)	0.96 (0.94, 0.99)	0.96 (0.93, 0.99)	0.99 (0.96, 1.02)
Oct. 2021 (-3)‡	0.84 (0.8, 0.87)	0.84 (0.8, 0.87)	0.84 (0.8, 0.88)	0.83 (0.8, 0.87)	0.84 (0.8, 0.87)	0.85 (0.82, 0.89)
Nov. 2021 (-2)‡	0.65 (0.6, 0.71)	0.65 (0.59, 0.71)	0.65 (0.59, 0.71)	0.64 (0.59, 0.7)	0.63 (0.58, 0.69)	0.67 (0.61, 0.73)
Dec. 2021 (-1)‡	0.47 (0.42, 0.53)	0.47 (0.42, 0.53)	0.47 (0.42, 0.53)	0.46 (0.41, 0.52)	0.46 (0.41, 0.52)	0.48 (0.43, 0.54)
Cardiovasc. Diseases	0.99 (0.94, 1.05)	0.96 (0.92, 1.01)	0.96 (0.92, 1.01)	0.96 (0.92, 1.01)	0.96 (0.92, 1.01)	0.96 (0.92, 1.01)
Diabetes Mellitus	0.94 (0.9, 0.99)	0.96 (0.91, 1.01)	1.01 (0.97, 1.05)	0.94 (0.9, 0.99)	0.95 (0.9, 0.99)	0.94 (0.9, 0.98)
Hypertension	0.99 (0.96, 1.03)	0.99 (0.96, 1.03)	0.94 (0.9, 0.99)	0.99 (0.96, 1.03)	0.99 (0.96, 1.03)	0.99 (0.96, 1.02)
COPD	0.89 (0.81, 0.98)	0.89 (0.81, 0.98)	0.89 (0.81, 0.98)	0.89 (0.8, 0.99)	0.89 (0.81, 0.98)	0.89 (0.81, 0.98)
Immunosuppression	0.95 (0.9, 1)	0.95 (0.9, 1)	0.95 (0.9, 1)	0.95 (0.9, 1)	0.95 (0.89, 1.01)	0.95 (0.9, 1)
Obesity (BMI ≥30)	0.95 (0.92, 0.98)	0.95 (0.92, 0.98)	0.95 (0.92, 0.98)	0.95 (0.92, 0.98)	0.95 (0.92, 0.98)	1.02 (0.98, 1.06)
Sep. 2021 and Comorbidity	0.88 (0.79, 0.99)	0.9 (0.8, 1.02)	0.94 (0.87, 1.01)	0.87 (0.63, 1.18)	1.02 (0.91, 1.14)	0.85 (0.8, 0.91)
Oct. 2021 and Comorbidity	1.05 (0.87, 1.27)	1.04 (0.85, 1.27)	1.01 (0.88, 1.16)	1.43 (0.87, 2.36)	0.85 (0.68, 1.06)	0.88 (0.79, 0.97)
Nov. 2021 and Comorbidity	0.71 (0.44, 1.12)	0.91 (0.51, 1.61)	0.99 (0.69, 1.43)	0.93 (0.26, 3.34)	1.46 (0.92, 2.31)	0.72 (0.53, 0.98)
Dec. 2021 and Comorbidity	0.96 (0.51, 1.84)	0.79 (0.35, 1.76)	0.69 (0.38, 1.26)	1.52 (0.38, 6.05)	1.44 (0.62, 3.35)	0.8 (0.55, 1.17)
p-value of the Likelihood Ratio Test of interaction vs without-interaction analyses	0.11	0.47	0.39	0.51	0.23	<0.01

(1) Reference period: administration of booster during August 2021

‡ Denotes the months prior to the outcome period. Therefore, those who received the booster in October 2021, received the third dose 3 months prior to the outcome period.

Table S4. GEE logistic regression results for SARS-Cov2 infection allowing for multiple negative tests

	Infection	
	OR	*CI(95%)(1)*
Exposure Groups/Booster period (2)		
Sep. 2021 (-4)‡	0.953	(0.932, 0.974)
Oct. 2021 (-3)‡	0.838	(0.814, 0.863)
Nov. 2021 (-2)‡	0.660	(0.623, 0.7)
Dec. 2021 (-1)‡	0.468	(0.434, 0.505)
Sex(Male)	1.097	(1.08, 1.114)
Age Groups(3)		
[30, 40)	0.801	(0.782, 0.821)
[40, 50)	0.826	(0.806, 0.846)
[50, 60)	0.829	(0.806, 0.852)
60+	0.706	(0.682, 0.73)
SES Group(4)		
High	1.006	(0.99, 1.023)
Low	0.932	(0.906, 0.959)
Other	0.779	(0.628, 0.966)
Social Sector(5)		
Arab	1.047	(0.998, 1.099)
Orthodox Jew	2.435	(2.334, 2.54)
Cardiovascular Diseases	0.964	(0.93, 0.999)
Diabetes Mellitus	0.977	(0.945, 1.011)
Hypertension	0.958	(0.934, 0.984)
COPD	0.896	(0.832, 0.966)
Immunosuppression	0.929	(0.894, 0.964)
Obesity (BMI ≥30)	0.936	(0.916, 0.958)
Test Calendar Week(6)		
Jan 8-14	2.393	(2.345, 2.441)
Jan 15-21	3.496	(3.427, 3.566)
Second Dose Month(7)		
Feb. 2021	1.236	(1.211, 1.261)
March 2021	1.270	(1.238, 1.303)
Apr. 2021	1.041	(0.98, 1.107)
May 2021	1.004	(0.895, 1.127)
June 2021	1.035	(0.854, 1.255)
July 2021	1.024	(0.829, 1.265)

In the analysis of VE against infection number of cases was 847,99 and controls was 374,617. (1) Robust 95% Wald confidence intervals were calculated using logistic generalized estimating equation (GEE) model, (2) Reference period: administration of booster during August 2021 (5 months prior to the outcome period), (3) Ref.: Age group [16, 30), (4) Ref.: High SES, (5) Ref.: Other, (6) Ref.: calendar week 01-07/01, (7) Ref.: January 2021. COPD: Chronic Obstructive Pulmonary Disease ‡ Denotes the months prior to the outcome period. Therefore, those who received the booster in October 2021, received the third dose 3 months prior to the outcome period.

Table S5. Conditional logistic regression results for SARS-CoV-2 infection including PCR tests taken up to 6 days after the fourth vaccine dose

	**Infection**	
	OR	*CI(95%)(1)*
Exposure Groups/Booster period(2)		
Sep. 2021 (-4)‡	0.974	(0.945, 1.004)
Oct. 2021 (-3)‡	0.846	(0.813, 0.881)
Nov. 2021 (-2)‡	0.690	(0.631, 0.755)
Dec. 2021 (-1)‡	0.471	(0.419, 0.529)
Cardiovascular Diseases	0.964	(0.924, 1.006)
Diabetes Mellitus	0.958	(0.92, 0.998)
Hypertension	0.988	(0.957, 1.02)
COPD	0.962	(0.886, 1.045)
Immunosuppression	0.908	(0.867, 0.951)
Obesity (BMI ≥30)	0.952	(0.925, 0.98)

In analysis of VE against infection number of Cases was 58,803 and Controls was 58,803
(1) 95% Wald confidence interval
(2) Reference period/exposure group: August 2021(5 months prior to the outcome period).
COPD: Chronic Obstructive Pulmonary Disease ‡ Denotes the months prior to the outcome period. Therefore, those who received the booster in October 2021, received the third dose 3 months prior to the outcome period.

Table S6. Additional analysis: adjusted Third Dose Effectiveness (VE) against SARS-Cov2 infection, including a separate stratum of days 0-6 after the fourth dose.

Exposure Groups/Booster period(1)	Adjusted Third Dose VE (%) for SARS-Cov2 Infection (CI 95%)-
Sep. 2021	4.7 (1.2, 8)
Oct. 2021	20.1 (16.2, 23.7)
Nov. 2021	40.3 (34.1, 45.8)
Dec. 2021	58.5 (53, 63.3)
4th Dose 0-6 Days	28.9 (24.4, 33.1)

(1) Reference period: administration of booster during August 2021
‡ Denotes the months prior to the outcome period. Therefore, those who received the booster in October 2021, received the third dose 3 months prior to the outcome period.

Table S7. Conditional logistic regression results for SARS-CoV-2 infection and COVID-19-related hospitalizations compared to second dose only vaccinees.

	Infection		SARS-Cov2 Related Hospitalization	
	OR	*CI(95%)(1)*	*OR*	*CI(95%)(1)*
Exposure Groups/Booster period(2)				
Aug. 2021 (-5)‡	0.840	(0.805, 0.877)	0.455	(0.239, 0.866)
Sep. 2021 (-4)‡	0.817	(0.788, 0.848)	0.450	(0.237, 0.853)
Oct. 2021 (-3)‡	0.709	(0.68, 0.739)	0.278	(0.124, 0.622)
Nov. 2021 (-2)‡	0.568	(0.522, 0.618)	0.508	(0.095, 2.706)
Dec. 2021 (-1)‡	0.406	(0.365, 0.451)	1.996	(0.275, 14.468)
Cardiovascular diseases	0.957	(0.914, 1.003)	2.454	(1.496, 4.027)
Diabetes Mellitus	0.950	(0.909, 0.992)	1.606	(0.936, 2.756)
Hypertension	1.000	(0.967, 1.034)	2.043	(1.304, 3.2)
COPD	0.910	(0.828, 0.999)	3.050	(1.283, 7.251)
Immunosuppression	0.937	(0.892, 0.983)	2.767	(1.644, 4.656)
Obesity (BMI ≥30)	0.951	(0.924, 0.979)	0.763	(0.484, 1.201)

In analysis of VE against infection number of Cases: 64,427and Controls:64,427

In analysis of VE against SARS-Cov2 related hospitalization number of Cases:361 and Controls:361

(1) 95% Wald confidence interval

(2) Reference period/exposure group: 2nd dose only.

COPD: Chronic Obstructive Pulmonary Disease ‡ Denotes the months prior to the outcome period. Therefore, those who received the booster in October 2021, received the third dose 3 months prior to the outcome period.

Table S8. Conditional logistic regression results for SARS-CoV-2 infection including PCR tests of individuals who also had a positive PCR test prior to August 2021.

	**Infection**	
	OR	*CI(95%)(1)*
Exposure Groups/Booster period(2)		
Sep. 2021 (-4)‡	0.96	(0.93, 0.99)
Oct. 2021 (-3)‡	0.84	(0.8, 0.87)
Nov. 2021 (-2)‡	0.65	(0.6, 0.71)
Dec. 2021 (-1)‡	0.48	(0.43, 0.54)
Cardiovascular Diseases	0.95	(0.9, 0.99)
Diabetes Mellitus	0.95	(0.91, 1)
Hypertension	0.99	(0.96, 1.03)
COPD	0.90	(0.82, 1)
Immunosuppression	0.94	(0.89, 0.99)
Obesity (BMI ≥30)	0.94	(0.91, 0.97)
Past infection (prior to Aug. 2021)	0.46	(0.35, 0.61)

In analysis of VE against infection number of Cases:55,590 and Controls:55,590
(1) 95% Wald confidence interval
(2) Reference period/exposure group: August 2021(5 months prior to the outcome period).
COPD: Chronic Obstructive Pulmonary Disease ‡ Denotes the months prior to the outcome period. Therefore, those who received the booster in October 2021, received the third dose 3 months prior to the outcome period.

Table S9. Additional analysis: adjusted Third Dose Effectiveness (VE) against SARS-Cov2 infection, including individuals who also had a positive PCR test prior to August 2021.

Exposure Groups/Booster period(1)	Adjusted Third Dose VE (%) for SARS-Cov2 Infection (CI 95%)
Sep. 2021	4.2 (1.2, 7)
Oct. 2021	16.5 (12.99, 19.8)
Nov. 2021	34.8 (28.83, 40.4)
Dec. 2021	51.8 (45.9, 57)

(1) Reference period: administration of booster during August 2021
‡ Denotes the months prior to the outcome period. Therefore, those who received the booster in October 2021, received the third dose 3 months prior to the outcome period.

Table S10. Comparison of exposure (time-since-vaccination) between the negative controls included in the analysis (third column) and the source population (the entire eligible MHS population, second column).

Exposure Period	Total Population	Controls (Tests)
Second Dose Only	189661(13.85%)	70570(15.85%)
Aug. 2021	510189(37.25%)	148821(33.43%)
Sep. 2021	446007(32.57%)	155489(34.93%)
Oct. 2021	168149(12.28%)	52237(11.73%)
Nov. 2021	31496(2.3%)	9509(2.14%)
Dec. 2021	23990(1.75%)	8561(1.92%)
