# Supplementary material for: Waning effectiveness of the third dose of the BNT162b2 mRNA COVID-19 vaccine
Source: Nat Commun. 2022 Jun 9;13:3203. doi: 10.1038/s41467-022-30884-6 (PMC9184525; doi:10.1038/s41467-022-30884-6)
Supplement: Supplementary file 2 — Reporting Summary [file 41467_2022_30884_MOESM2_ESM.pdf]

nature portfolio

Corresponding author(s): Tal Patalon
Last updated by author(s): May 11, 2022

# Reporting Summary

Nature Portfolio wishes to improve the reproducibility of the work that we publish. This form provides structure for consistency and transparency in reporting. For further information on Nature Portfolio policies, see our Editorial Policies and the Editorial Policy Checklist.

## Statistics

For all statistical analyses, confirm that the following items are present in the figure legend, table legend, main text, or Methods section.

| n/a | Confirmed | |
| --- | --- | --- |
| ☐ | ☒ | The exact sample size (*n*) for each experimental group/condition, given as a discrete number and unit of measurement |
| ☐ | ☒ | A statement on whether measurements were taken from distinct samples or whether the same sample was measured repeatedly |
| ☐ | ☒ | The statistical test(s) used AND whether they are one- or two-sided<br>*Only common tests should be described solely by name; describe more complex techniques in the Methods section.* |
| ☐ | ☒ | A description of all covariates tested |
| ☐ | ☒ | A description of any assumptions or corrections, such as tests of normality and adjustment for multiple comparisons |
| ☐ | ☒ | A full description of the statistical parameters including central tendency (e.g. means) or other basic estimates (e.g. regression coefficient) AND variation (e.g. standard deviation) or associated estimates of uncertainty (e.g. confidence intervals) |
| ☐ | ☒ | For null hypothesis testing, the test statistic (e.g. *F*, *t*, *r*) with confidence intervals, effect sizes, degrees of freedom and *P* value noted<br>*Give P values as exact values whenever suitable.* |
| ☒ | ☐ | For Bayesian analysis, information on the choice of priors and Markov chain Monte Carlo settings |
| ☒ | ☐ | For hierarchical and complex designs, identification of the appropriate level for tests and full reporting of outcomes |
| ☒ | ☐ | Estimates of effect sizes (e.g. Cohen's *d*, Pearson's *r*), indicating how they were calculated |

*Our web collection on statistics for biologists contains articles on many of the points above.*

## Software and code

Policy information about availability of computer code

Data collection: *Provide a description of all commercial, open source and custom code used to collect the data in this study, specifying the version used OR state that no software was used.*

Data analysis: *Provide a description of all commercial, open source and custom code used to analyse the data in this study, specifying the version used OR state that no software was used.*

For manuscripts utilizing custom algorithms or software that are central to the research but not yet described in published literature, software must be made available to editors and reviewers. We strongly encourage code deposition in a community repository (e.g. GitHub). See the Nature Portfolio guidelines for submitting code & software for further information.

## Data

Policy information about availability of data

All manuscripts must include a data availability statement. This statement should provide the following information, where applicable:

- Accession codes, unique identifiers, or web links for publicly available datasets
- A description of any restrictions on data availability
- For clinical datasets or third party data, please ensure that the statement adheres to our policy

According to the Israel Ministry of Health regulations, individual-level data cannot be shared openly. Specific requests for remote access to de-identified community-level data should be referred to KSM, Maccabi Healthcare Services Research and Innovation Center.

# Field-specific reporting

Please select the one below that is the best fit for your research. If you are not sure, read the appropriate sections before making your selection.

- [x] Life sciences
- [ ] Behavioural & social sciences
- [ ] Ecological, evolutionary & environmental sciences

For a reference copy of the document with all sections, see nature.com/documents/nr-reporting-summary-flat.pdf

# Life sciences study design

All studies must disclose on these points even when the disclosure is negative.

| | |
|---|---|
| Sample size | The study population included ALL MHS members aged 16 or older who received at least two doses of the BNT162b2 vaccine by August 1, 2021. |
| Data exclusions | Individuals were excluded from the study if they had a positive SARS-CoV-2 polymerase chain reaction (PCR) assay test result prior to the start of the outcome period, disengaged from MHS for any reason prior to the start of the study period or joined MHS after to March 2020, hence might have an incomplete COVID-19-related history. |
| Replication | The analyses were replicated twice in our internal team using the same data successfully. |
| Randomization | Not relevant for this study, as this was a retrospective data study, utilizing an existing database, where all participant who met the inclusion criteria were included. |
| Blinding | Not relevant for this study, as this was an observational retrospective data study, where the researchers explored all participant who met the inclusion criteria, and no intervention was done. |

# Reporting for specific materials, systems and methods

We require information from authors about some types of materials, experimental systems and methods used in many studies. Here, indicate whether each material, system or method listed is relevant to your study. If you are not sure if a list item applies to your research, read the appropriate section before selecting a response.

## Materials & experimental systems

| n/a | Involved in the study | |
|---|---|---|
| x | | Antibodies |
| x | | Eukaryotic cell lines |
| x | | Palaeontology and archaeology |
| x | | Animals and other organisms |
| | x | Human research participants |
| x | | Clinical data |
| x | | Dual use research of concern |

## Methods

| n/a | Involved in the study | |
|---|---|---|
| x | | ChIP-seq |
| x | | Flow cytometry |
| x | | MRI-based neuroimaging |

# Human research participants

Policy information about studies involving human research participants

| | |
|---|---|
| Population characteristics | The study population included all MHS members aged 16 or older who received at least two doses of the BNT162b2 vaccine by August 1, 2021. Individuals were excluded from the study if they had a positive SARS-CoV-2 polymerase chain reaction (PCR) assay test result prior to the start of the outcome period, disengaged from MHS for any reason prior to the start of the study period or joined MHS after to March 2020, hence might have an incomplete COVID-19-related history.<br><br>Individual-level demographic data of the study population included sex, age and a coded residential socioeconomic geographical statistical area (GSA), assigned by Israel's Central Bureau of Statistics, which is the smallest geostatistical unit of the Israeli census (corresponds to neighborhoods) and embodies the socioeconomic status. COVID-19 related data consisted of dates of vaccination and results of any polymerase chain reaction (PCR) tests for SARS-CoV-2, given that all such tests are recorded centrally. PCR tests were readily available during the outcome period, for reasons including clinical symptoms, suspected exposure to infected individuals, public events requirements and more. COVID-19-related hospitalizations and mortality records were retrieved as well. Captured data also consisted of information on chronic diseases from MHS' automated registries, including cardiovascular diseases21, hypertension, diabetes22, chronic kidney disease (CKD)23, chronic obstructive pulmonary disease (COPD), obesity (defined as a body mass index of 30 or higher) and immunocompromised conditions.<br><br>546,924 PCR tests by 389,265 eligible MHS members were performed during the outcome period of January 1 to January 21, 2022. 53,486 tests (performed by 38,145 individuals) were excluded from the main analysis, as they were conducted after the administration of the fourth dose (see additional analyses). Population characteristics can be found in Table 1. Overall, those vaccinated early were more chronically ill, likely correlating with earlier compliance to vaccination by the older population, emphasizing the need for adjustment. |
| Recruitment | A retrospective study; no active recruitment was involved |
| Ethics oversight | This study was approved by the MHS (Maccabi Healthcare Services) Institutional Review Board. Due to the retrospective design of the study, informed consent was waived by the IRB, as all identifying details of the participants were removed before computational analysis. |

Note that full information on the approval of the study protocol must also be provided in the manuscript.
